# Transient nature of cooperation by pay-it-forward reciprocity

**DOI:** 10.1038/srep19471

**Published:** 2016-01-20

**Authors:** Yutaka Horita, Masanori Takezawa, Takuji Kinjo, Yo Nakawake, Naoki Masuda

**Affiliations:** 1 National Institute of Informatics, 2-1-2 Hitotsubashi, Chiyoda-ku, Tokyo 101-8430, Japan; 2 JST, ERATO, Kawarabayashi large graph project, c/o Global Research Center for Big Data Mathematics, NII, 2-1-2 Hitotsubashi, Chiyoda-ku, Tokyo 101-8430, Japan; 3 Department of Behavioral Science, Hokkaido University, N10W7, Kita-ku, Sapporo, 060-0810, Japan; 4 Center for Experimental Research in Social Sciences, Hokkaido University, N10W7, Kita-ku, Sapporo, 060-0810, Japan; 5 Department of Engineering Mathematics, University of Bristol, Merchant Venturers Building, Woodland Road, Clifton, Bristol BS8 1UB, United Kingdom

## Abstract

Humans often forward kindness received from others to strangers, a phenomenon called the upstream or pay-it-forward indirect reciprocity. Some field observations and laboratory experiments found evidence of pay-it-forward reciprocity in which chains of cooperative acts persist in social dilemma situations. Theoretically, however, cooperation based on pay-it-forward reciprocity is not sustainable. We carried out laboratory experiments of a pay-it-forward indirect reciprocity game (i.e., chained gift-giving game) on a large scale in terms of group size and time. We found that cooperation consistent with pay-it-forward reciprocity occurred only in a first few decisions per participant and that cooperation originated from inherent pro-sociality of individuals. In contrast, the same groups of participants showed persisting chains of cooperation in a different indirect reciprocity game in which participants earned reputation by cooperating. Our experimental results suggest that pay-it-forward reciprocity is transient and disappears when a person makes decisions repeatedly, whereas the reputation-based reciprocity is stable in the same situation.

In December 2012, a customer generously paid for coffee for the next stranger customer in a Tim Hortons drive-through in Winnipeg. Subsequent customers did the same act such that a chain of generous behavior eventually involved 228 customers^1^. Similar phenomena have been witnessed in other shops^2^,^3^,^4^,^5^. This form of cooperative behavior, cooperating with a stranger after receiving cooperation from a different individual, is referred to as pay-it-forward reciprocity, also known as upstream reciprocity and generalized reciprocity. Pay-it-forward reciprocity has been observed in humans in some behavioral experiments^6^,^7^,^8^,^9^,^10^,^11^ and fields^12^, and also in rats^13^.

Pay-it-forward reciprocity is one of the two forms of indirect reciprocity, in which one would cooperate with strangers even when not to cooperate is apparently more lucrative (i.e., social dilemma situations). Indirect reciprocity is considered to enable large-scale cooperation in a population in which it is practically impossible for all individuals to be acquainted with each other^14^,^15^. Theoretically, cooperation under pay-it-forward reciprocity is stable only when it is combined with other stand-alone mechanisms of cooperation such as direct reciprocity (i.e., repeated interaction)^16^, spatial networks^16^, heterogeneous networks^17^, networks with community structure or sparse networks^18^, small population size^19^,^20^,^21^, mobility of players^22^, assortative interaction among players^23^, and a population variant of tit-for-tat^24^. The reputation-based reciprocity is the other form of indirect reciprocity in which those who have cooperated receive a good reputation and individuals would cooperate with those with good reputations. Reputation-based cooperation has also been widely observed in humans in laboratory experiments^25^,^26^,^27^,^28^ and online society^29^,^30^, and also in cleaner fish^31^,^32^. However, differently from pay-it-forward reciprocity, reputation-based indirect reciprocity has strategic rationality and is theoretically stable as payoff-maximizing behavior^14^,^33^,^34^.

In this paper, contrary to the previous empirical studies^6^,^7^,^8^,^9^,^10^,^11^,^12^,^35^,^36^,^37^,^38^,^39^, we demonstrate that the ability of pay-it-forward reciprocity to produce a chain of good-will on a large scale is fairly limited as compared to that of reputation-based reciprocity. In particular, we show that a chain of cooperation sustained by pay-it-forward reciprocity, but not by reputation-based reciprocity, is transient when an individual is made to act repeatedly. To this end, we carried out laboratory experiments in which each participant was engaged in long chains of behavior in two types of indirect reciprocity games. The participants submitted decisions many times, and we observed dynamics of their behavior. Chains of cooperation sustained by reputation-based reciprocity are expected to emerge robustly in such a situation because it is payoff-maximizing behavior^14^,^33^,^34^. On the contrary, we postulate that a chain of good-will counting on pay-it-forward reciprocity, which is not strategically rational behavior^16^,^17^,^18^,^19^,^20^,^21^,^22^,^23^,^24^, will gradually disappear as participants accumulate decisions over time.

## Results

### Time series of cooperation in the two indirect reciprocity games

**Figure 1:**
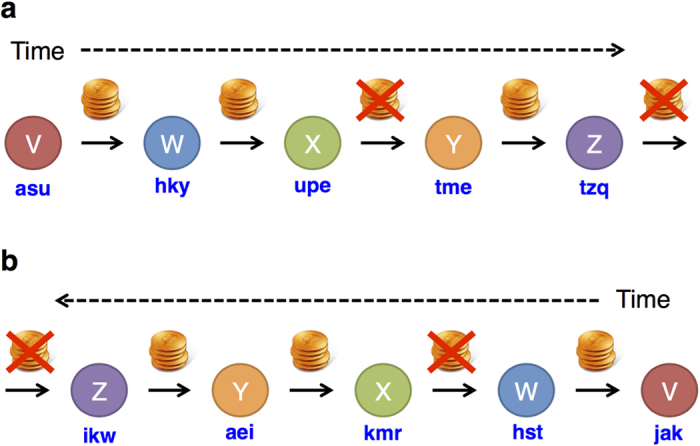
Schematic of the indirect reciprocity games. (**a**) Pay-it-forward game. (**b**) Reputation-based game. Each participant received random three-letter names.

Participants played chained versions of the pay-it-forward reciprocity game and reputation-based reciprocity game in a group of 17 or 19 participants. In both games, each participant either donated (i.e., cooperate, denoted by C) or did not donate (i.e., defect, denoted by D) the money received by the experimenter to the downstream neighbor in the chain (Fig. 1). If the participant selected C, he/she lost the money and the neighbor received the doubled amount of money. If the participant selected D, he/she kept the money and the neighbor received nothing. The participants made decisions many times (maximum 45 times per participant) in each of the two games in anonymous situations. We refer to each decision opportunity as the round; for example, round three indicates that each participant submits a decision for the third time. In the pay-it-forward game, each participant (except for the first participant in a chain) was informed of the previous decision made by the upstream neighbor toward the participant (Fig. 1(a)). In the reputation-based game, each participant was informed of the decision made by the downstream neighbor toward its downstream neighbor (Fig. 1(b)). See Methods for more details about the experimental procedure.

To examine the possibility of indirect reciprocity, we measure three types of probability of cooperation in each game. First, *p*(C) denotes the fraction of cooperation, which may depend on the participant and round. Second, *p*(C|C) and *p*(C|D) denote the fraction of cooperation right after the participant is informed of the C and D decisions of the previous participant, respectively. It should be noted that *p*(C|C) and 1 − *p*(C|D) quantify the amount of positive and negative indirect reciprocity, respectively.

**Figure 2:**
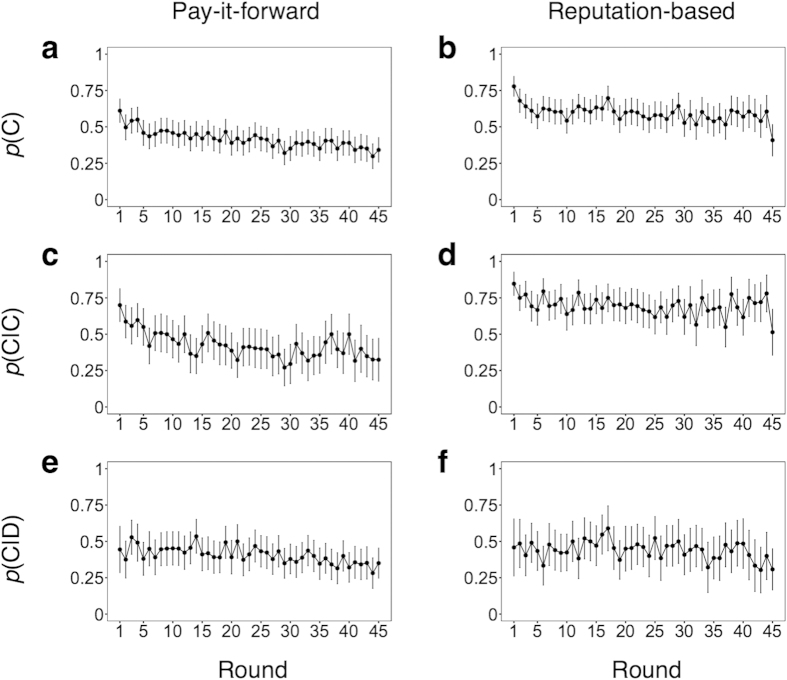
Time courses of the fraction of cooperation averaged over all participants. A round corresponds to one decision made by each participant (e.g., the third round implies the third decision made by each participant in the entire game). (**a**) *p*(C) in the pay-it-forward game, (**b**) *p*(C) in the reputation-based game, (**c**) *p*(C|C) in the pay-it-forward game, (**d**) *p*(C|C) in the reputation-based game, (**e**) *p*(C|D) in the pay-it-forward game, and (**f**) *p*(C|D) in the reputation-based game. The error bars represent 95 % confidence intervals (±1.96 × SE).

The probability of cooperation in each round averaged over all participants is shown in Fig. 2. In the pay-it-forward game, both unconditional (Fig. 2(a)) and conditional (Fig. 2(c,e)) probabilities of cooperation seem to decline over rounds, particularly in early rounds. However, in the reputation-based game, the probability of both unconditional (Fig. 2(b)) and conditional (Fig. 2(d,f)) cooperation is larger and stable over time.

Figure 2(b,d) indicate that *p*(C) and *p*(C|C) dropped in the last round in the reputation-based game, respectively. This is because, in this game, participants who played in the last position in a chain of decisions were informed before making a decision that no upstream neighbor existed.

### Determinants of cooperation in the two games - Statistical analysis

To examine patterns of indirect reciprocity in pay-it-forward and reputation-based games, we conducted multivariate generalized linear mixed model (GLMM) analysis. Behavior was defined to be the sole binary dependent variable (D = 0, C = 1). We adopted the following three independent variables and their interactions. The decision of the previous participant as a binary variable (D = 0, C = 1), the round as an integer valued variable (ranging from 1 to 45), and the game type as a binary variable (reputation-based game = 0, pay-it-forward game = 1). Indirect reciprocity is represented as the effect of the decision of the previous participant on the behavior of the current participant. If the indirect reciprocity is weakened or strengthened over time, the two-way interaction effect between the round and the decision of the previous participant should be significant. If the patterns of the indirect reciprocity differ between the two types of game, two- or three-way interaction effects involving the game type and the decision of the previous participant should be significant. Two more independent variables were entered as control variables. The first additional variable was the degree of pro-sociality, so-called social value orientation (SVO; pro-self = 0, pro-social = 1)^40^,^41^,^42^, which was determined by the questionnaires that the participants had gone through 1–2 months before the experiment (see Methods and Supplementary Methods for details). The second additional variable was the gender (female = 0, male = 1). The participant's ID (categorical values assigned to each participant, ranging from 1 to 131) was used as a random effect affecting the intercept. We assumed a binomial distribution with a logistic link function.

First, to examine whether the patterns of indirect reciprocity vary between the two types of game, we constructed 16 different models in which each of the four possible two- or three-way interaction terms was switched on or off. The four possible interaction terms were “decision of the previous participant x round”, “decision of the previous participant x game type”, “round x game type”, and “decision of the previous participant x round x game type”. We carried out the GLMM analysis for each of the 16 models and compared the values of Akaike’s information criterion (AIC) (Supplementary Table S2). The best model (AIC = 9035.7) included the two two-way interaction effects except the “decision of the previous participant x round” and also included the three-way interaction effect. The three-way interaction (i.e., decision of the previous participant x round x game type) was significant (odds ratio = .99, *P* = 0.01; Supplementary Table S3) in the best model. This result suggests that the patterns of indirect reciprocity vary between the two types of game.

Next, to reveal the difference between the two games, we conducted GLMM analysis separately for each type of game, where “decision of the previous participant x round” was the sole two-way interaction term. The results are shown in Table 1. The two-way interaction was significant in the pay-it-forward game but not in the reputation-based game. Furthermore, in the pay-it-forward game, we obtained the following results. First, cooperation significantly decreased as the round progressed. Second, if we neglected the effect of the round, the decision of the previous participant did not significantly affect the current participant’s decision. Third, the pro-sociality of participant significantly increased the probability of cooperation compared to the pro-selves. Fourth, the participant's gender did not affect behavior. Separate analysis indicated that *p*(C|C) was significantly larger than *p*(C|D) in the first and second rounds but they were not different in the subsequent rounds in the pay-it-forward game (Supplementary Table S4). Pay-it-forward reciprocity behavior was only significant in the first two rounds. This result is consistent with casual observations made with Fig. 2(a,c,e) and with the presence of the “decision of the previous participant x round” two-way interaction (Table 1). Taken together, cooperation as pay-it-forward reciprocity is a transient phenomenon.

In contrast to the pay-it-forward game, in the reputation-based game, the probability of cooperation when the previous player had cooperated was significantly larger than that when the previous player had defected (Table 1). Separate analysis confirmed that *p*(C|C) was significantly larger than *p*(C|D) in most of the rounds in the reputation-based game (Supplementary Table S4). These results are consistent with those shown in Fig. 2(b,d,f), which show that *p*(C|C) is larger than *p*(C|D) throughout the experiment. Therefore, reputation-based indirect reciprocity behavior (i.e., C after observing C and D after observing D) was persistent over time in our experiments. Pro-sociality did not affect the probability to cooperate. Males cooperated more than females, but this effect was marginal. The effect of the round was insignificant, consistent with observations made with Fig. 2(b,d,f).

Two remarks are in order. First, for each type of game, we also analyzed the univariate GLMM to find that the results were consistent with those for the multivariate GLMM shown in Table 1; the results for the univariate analysis are shown in Supplementary Table S5. Second, in fact, the participants in the same group influenced each other such that responses of different participants in the same group may be correlated. We confirmed that all the results held true even when the group was added as a second random effect in the GLMMs (Supplementary Table S6).

To strengthen our conclusions, we also compared *p*(C), *p*(C|C), and *p*(C|D) aggregated over the rounds and participants between the two games. We conducted three GLMM analyses. In each analysis, *p*(C), *p*(C|C), or *p*(C|D) was used as a dependent variable, the game type (reputation-based game = 0, pay-it-forward game = 1) as an independent variable, and both the individual and the group as random effects, assuming the normal distribution. The effect of game type was significant when we used *p*(C), or *p*(C|C) as the dependent variable (*p*(C): coefficient = −0.20, *P* < 0.01; *p*(C|C): coefficient = −0.29, *P* < 0.01). Both *p*(C) and *p*(C|C) were significantly larger in the reputation-based game than in the pay-it-forward game (*p*(C), pay-it-forward: *Mean* = 0.42, *SD* = 0.34; reputation-based: *Mean* = 0.62, *SD* = 0.32; *p*(C|C), pay-it-forward: *Mean* = 0.42, *SD* = 0.38; reputation-based: *Mean* = 0.71, *SD* = 0.34). However, the effect of game type was not significant when we used *p*(C|D) as the dependent variable (coefficient = −0.05, *P* = 0.15). We found no significant difference between *p*(C|D) in the pay-it-forward game and that in the reputation-based game (pay-it-forward: *Mean* = 0.42, *SD* = 0.39; reputation-based: *Mean* = 0.47, *SD* = 0.38).

Finally, we examined the degree of reciprocity defined by


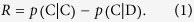


A participant with a large *R* value tends to show both positive reciprocity (C after C) and negative reciprocity (D after D). We conducted a GLMM analysis using the *R* value as a dependent variable and the game type as an independent variable, and found that the effect of game was significant (coefficient = −0.23, *P* < 0.01). The *R* value averaged over the participants was significantly larger in the reputation-based than the pay-it-forward game (pay-it-forward: *Mean* = 0.004, *SD* = 0.38; reputation-based: *Mean* = 0.23, *SD* = 0.33).

In summary, cooperation as pay-it-forward reciprocity quickly declines over time. With the data aggregated over time, we have found that cooperation in the pay-it-forward reciprocity does not occur as indirect reciprocity but is sustained by pro-sociality of the participant. We also tested effects of pro-sociality of the participants on pay-it-forward reciprocity to find that the “decision of the previous participant x SVO” two-way interaction was only marginally significant (Supplementary Table S7). Therefore, pro-sociality was not considered to cause pay-it-forward behavior. In contrast to the pay-it-forward game, cooperation in the reputation-based game was persistent and occured as indirect reciprocity.

### Dependence on participants

**Figure 3:**
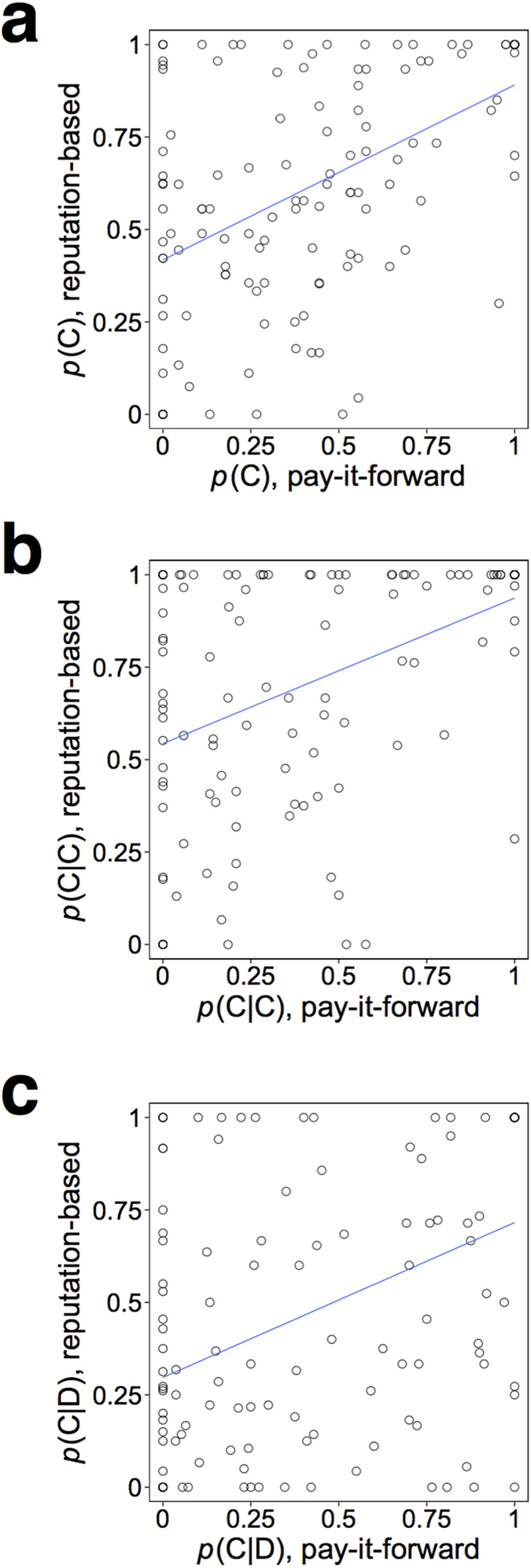
The relationship between the probability of cooperation in the pay-it-forward game and that in the reputation-based game. A circle represents a participant. The solid lines represent the linear regression. (**a**) *p*(C), (**b**) *p*(C|C), and (**c**) *p*(C|D).

To take a closer look at individual differences in the observed behavior, we calculated probabilities of cooperation for each participant and examined correlation of the probabilities between the two games. The relationship between *p*(C) in the pay-it-forward game, calculated for each participant, and *p*(C) in the reputation-based game is shown in Fig. 3(a). A circle represents a participant. The *p*(C) value is positively correlated between the two games (Pearson’s *r* = 0.50, *N* = 131, *P* < 0.01).

We found that *p*(C|C) and *p*(C|D) were also correlated between the two games (*p*(C|C): Fig. 3(b), Pearson’s *r* = 0.44, *N* = 131, *P* < 0.01; *p*(C|D): Fig. 3(c), Pearson’s *r* = 0.42, *N* = 131, *P* < 0.01). However, each of *p*(C|C) and *p*(C|D) was not correlated between the two games when we regressed out the effect of *p*(C) (*p*(C|C): Pearson’s *r* = 0.07, *N* = 131, *P* = 0.43; *p*(C|D): Pearson’s *r* = 0.12, *N* = 131, *P* = 0.19; see Methods for the definition). Therefore, the degree of reciprocal cooperation was not correlated between the two games when the effect of unconditional probability of cooperation (i.e., *p*(C)) was controlled away. Consistent with these results, the *R* values calculated separately for each participant were not significantly correlated between the two games (*r* = 0.10, *N* = 131, *P* = 0.25; Supplementary Fig. S2).

### Contagion of cooperation and defection

Previous empirical studies and field observations suggested that cooperative or defective behavior was contagious^1^,^2^,^3^,^4^,^5^,^9^,^11^,^43^. To address this issue within our data, we modeled successions of cooperation and those of defection on the chain of participants as simple stochastic processes that neglected individuality of the participants. We define the length of cooperation as the number of C that successively occurs before a D occurs. For example, if a participant that received/observed defection cooperates, the next two participants also cooperate, and the subsequent participant defects (i.e., …DCCCD…), the length of C is equal to three. The length of defection is defined in the same manner.

**Figure 4:**
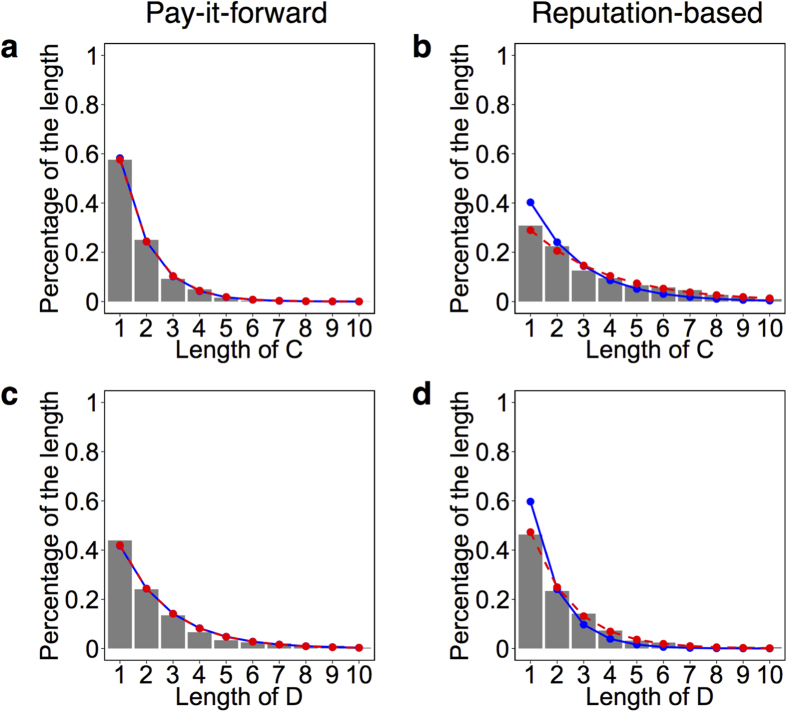
Frequencies of the length of cooperation and that of defection. The bars represent empirical frequencies. There were some samples that had run lengths larger than ten and hence were not shown in each panel. The solid and dashed curves represent the geometric distributions estimated based on the i.i.d. and Markov assumptions, respectively. (**a**,**b**) show the frequencies of the length of C in the pay-it-forward game and those in the reputation-based game, respectively. In (**a**), both solid and dashed lines are based on *p* ≈ 0.42. In (**b**), the solid line is based on *p* = 0.60, and the dashed line is based on *p* = 0.71. (**c**,**d**) show the frequencies of the length of D in the pay-it-forward game and those in the reputation-based game, respectively. In (**c**), both solid and dashed lines are based on *p* ≈ 0.58. In (**d**), the solid line is based on *p* = 0.40, and the dashed line is based on *p* = 0.53. The solid and dashed lines almost completely overlap in (**a**,**c**).

The frequencies of the length of C and that of D are shown for each type of game in Fig. 4. If the decision is independent of the participant and round (i.e., independently and identically distributed, or i.i.d.), the length of C and that of D obey the geometrical distribution given by


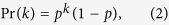


where *k* is the length of C or D. The maximum likelihood estimator for the length of C and D is given by 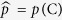 and 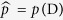, respectively. The estimated geometric distributions are drawn by the solid lines in Fig. 4 (hidden under the dashed lines in Fig. 4(a,c)). Figure 4(a,c) correspond to the length of C and D in the pay-it-forward game, respectively. In these figures, the empirical distributions did not significantly deviate from the geometrical distributions given by equation (2) (length of C:, KS test, *D* = 0.01, *P* = 1.00; length of D: KS test, *D* = 0.02, *P* = 0.89). Therefore, the results do not support contagion of cooperation or defection beyond the expectation of i.i.d. sequences. In contrast, in the reputation-based game, the empirical distribution of the length of C and that of D, shown by the bars in Fig. 4(b,d), respectively, significantly deviated from those of the geometrical distributions shown by the solid lines (length of C: KS test, *D* = 0.13, *P* < 0.01; length of D: KS test, *D* = 0.14, *P* < 0.01). Therefore, the succession of C and D observed in the reputation-based game is not explained under the assumption of independent decision making.

Next, we considered a second model in which the participants independently use participant-independent conditional probabilities *p*(C|C) and *p*(C|D) to make decisions. Under this assumption, the decisions are generated by the Markov chain with two states, C and D (Supplementary Fig. S5). The theoretical distribution of the length of C and that of D in this case is given by the geometrical distribution (equation (2)) with 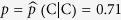 and 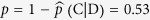, respectively (Supplementary Methods). The geometrical distributions estimated on the basis of the two-state Markov chain are shown by the dashed lines in Fig. 4. In the pay-it-forward game, the two-state Markov chain (dashed lines) and the i.i.d. model (solid lines) yielded indistinguishable results. In the reputation-based game, for both the length of C and that of D, the geometrical distributions derived from the two-state Markov chain (dashed lines) were different from those derived from the i.i.d. model (solid lines), and the former distributions captured the empirical distributions within insignificant error (length of C: KS test, *D* = 0.04, *P* = 0.55; length of D: KS test, *D* = 0.03, *P* = 0.91). Therefore, we conclude that indirect reciprocity behavior, which corresponds to the two-state Markov chain, is necessary for explaining succession of C and D observed in the reputation-based game.

## Discussion

We investigated the effects of two types of indirect reciprocity – pay-it-forward and reputation-based reciprocity – on contagion of cooperation. The participants were embedded in long chains and made decisions many times toward anonymous others. In the reputation-based game, the fraction of cooperation was relatively large and stable across rounds. Cooperation of previous participants significantly increased the focal player’s cooperation, consistent with previous theoretical^14^,^33^,^34^ and experimental results^25^,^26^,^27^,^28^. Even selfish (pro-self) players tended to cooperate to maintain their reputations. This behavioral correlation (i.e., C after C and D after D) resulted in longer successions of C and D in chains of participants than in the hypothetical case in which participants independently made decisions. In the pay-it-forward game, we did not find reciprocal behavior or successions of C and D longer than expected from the case of independent decisions. This result is consistent with theoretical results indicating that cooperation based on pay-it-forward reciprocity is not sustainable on its own^15^,^16^,^17^,^18^,^19^,^20^,^21^,^22^,^23^,^24^,^44^. Exclusively in this game, the pro-sociality of participants significantly promoted cooperation. Cooperation and its ostensible contagion were sustained by inclination of some participants to unconditional cooperation, not by reciprocity. The fraction of cooperation also declined over rounds in the pay-it-forward game.

Much of extant empirical evidence is in favor of pay-it-forward reciprocity. In fact, such evidence does not contradict the present results. First, each participant submitted a decision just once in many previous experiments^6^,^7^,^8^,^10^,^36^,^45^ and field observations^1^,^2^,^3^,^4^,^5^ showing pay-it-forward reciprocity. The participants in the present study also showed pay-it-forward reciprocity behavior in the first two rounds.

Second, pay-it-forward reciprocity was found to endure even when the same participants made decisions repeatedly in previous experiments^35^,^37^. In these experiments, participants were formed in fixed short cycles composed of three or four individuals. In this situation, the participants might have felt that their cooperative actions would be reimbursed through chained reactions of cooperation, which is consistent with the theoretical result that pay-it-forward reciprocity is viable on short cycles^19^,^20^,^21^. Cooperation on the basis of pay-it-forward reciprocity in small groups is a strategically sensible choice. In contrast, we used groups of 17 or 19 anonymized participants that were placed on chains. Therefore, the participants would not be able to feel that they were embedded in short cycles. Under this circumstance, pay-it-forward reciprocity occurred only in initial rounds. However, we do not exclude the possibility that our group size is still too small to observe significant pay-it-forward behavior. The participants may have felt that they were playing in a relatively small group. Then, they may have tried to act strategically, not in a pay-it-forward manner.

Third, two other experimental studies revealed behavior similar to pay-it-forward reciprocity. Fowler and Christakis reported contagion of cooperation in the public goods game where participants interacted in groups that were randomly and independently created in different rounds^9^. Grujić *et al.* conducted experiments with the prisoner’s dilemma game and a random matching protocol to find considerable cooperation under some conditions (i.e., when the focal participant cooperated last time)^46^. These results seem to contradict our argument that pay-it-forward reciprocity is unstable in a long run. Although unclear, we postulate that the difference has arisen from the fact that a participant has interacted with multiple others in a round in the previous experiments. In every round, a participant was engaged in the public goods game with three other participants^9^ or the prisoner’s dilemma game with each of four other participants^46^. In contrast, participants in our experiment observed the behavior of only a single peer in every round.

We are not the first to report instability of pay-it-forward reciprocity. Experiments conducted on Amazon Mechanical Turk yielded no^47^ or weak^11^ evidence of pay-it-forward reciprocity. High sensitivity of the degree of pay-it-forward reciprocity to experimental methods reported in a previous study^8^ is also consistent with instability of pay-it-forward reciprocity. Relative to these studies, the contribution of the present study is to have shown that cooperation as pay-it-forward reciprocity quickly decays in time and pro-sociality, not reciprocity, drives cooperation.

We showed that reputation-based reciprocity was more stable than pay-it-forward reciprocity. In fact, reputation-based reciprocity needs a system to monitor behavior of members and share their reputations in the community^48^,^49^,^50^. Pay-it-forward reciprocity, however, does not require such a system. Therefore, pay-it-forward reciprocity may remain a viable mechanism to yield cooperation, though transient, when reputation management systems are unavailable.

Since when Adam Smith^51^ and David Hume^52^ discussed roles of moral emotions as a basis of moral judgments and behavior, emotions such as empathy, shame, guilt, and gratitude, are argued to be a core proximate mechanism underlying human cooperation^53^,^54^. In particular, cooperation in one-shot and anonymous situations, which does not provide any material benefit to actors, may be driven by moral^55^ or positive emotions^56^, intervening cost-benefit calculus possibly made by actors. Emotions are also contagious^57^. Positive emotion of gratitude may strengthen social relationships^58^,^59^ and play a key role in nurturing strong bonds in a society^60^,^61^. With these lines of evidence combined, a possible interpretation of the present results on pay-it-forward reciprocity is that it occurs via positive emotions. Previous psychological^7^,^45^,^60^,^61^,^62^ and neuroimaging^63^ studies showed that cooperation occurring as pay-it-forward reciprocity or contagion was induced by positive emotions such as gratitude and empathy. This interpretation is consistent with a transient nature of pay-it-forward reciprocity shown in the present study; positive emotions were shown to be transient in other experiments using different paradigms^62^. Moral emotions undoubtedly underlie moral behavior of humans. However, a chain of one-shot cooperation sustained by moral emotions may be fragile when people frequently make decisions. It is an important question to examine limits and potentials of moral emotions as a fundament of cooperative human society.

## Methods

### Ethics statements

The present research was approved by the ethic committee of the National Institute of Informatics, Japan and the Center for Experimental Research in Social Sciences at Hokkaido University, Japan. Participants read and signed informed consent forms before participating. The experiments were carried out in accordance with the approved guideline.

### Participants

Participants were 131 undergraduate students at Hokkaido University in Japan. They were recruited from a large participant pool via e-mail. The participants received monetary rewards depending on the performance in the game. Participants formed a group of 17 or 19 participants, and there were seven groups. Supplementary Table S1 shows detailed information about individual groups.

### Procedures

Upon arrival, participants were escorted into a lecture room which had a seating capacity of at least 30 people. Each participant sat in front of a tablet computer. The partitions between adjacent participants prohibited them from seeing each other’ face and the display of the tablet computer. Once all participants sat, an experimenter gave instructions about the experiment using audible slides. The participants also received a written summary of the instructions, which was put on the desk during the experiment. They were told that the anonymity of their decisions was secured throughout the experiment.

Each participant played both the pay-it-forward and the reputation-based games. The order of games was counterbalanced. Before each game started, the participants answered a questionnaire regarding the rule of the games. Those answering incorrectly were led to the correct answers by the experimenter, who mentioned the corresponding part of the written instructions to the participants. Each type of game started after all participants answered the questions correctly after possible corrections. After finishing all rounds of both games, the participants were individually paid according to the results of the two games. A participant received 1,236 Japanese yen (about 9.9 US dollars; 1 yen ≈ 0.008 US dollars) on average. It took about 90 minutes for a participant to finish both games.

### Games

The participants played two types of chained gift-giving games. Five pairs of participants were randomly selected from the group without overlap. One participant in each pair was assigned to a role of donor, and the other to a role of recipient with the equal probability (i.e., a half). The donor was given 10 yen (about 8 US cents) from the experimenter and then decided whether or not to donate it. If the donor donated, he/she was left with no money, whereas the recipient received the doubled amount (i.e., 20 yen). If the donor did not donate, he/she kept 10 yen, and the recipient did not receive anything. The participants submitted decisions through a tablet PC (Windows Surface Pro 2, Microsoft 256 GB, 94X-00012). The PCs were connected via a Wi-Fi network. The experimental software was developed using z-Tree^64^.

The participants were embedded in a chain and sequentially made decisions. Figure 1(a) schematically represents the decision flow in the pay-it-forward game. Player X is informed of player W’s decision (donate the 10 yen, i.e., C, or do not donate, i.e., D). Then, player X makes a decision toward player Y, X’s decision is revealed to Y, Y makes a decision toward Z, and so on. Figure 1(b) represents the decision flow in the reputation-based game. Player X is informed of W’s decision toward V. Then, X makes a decision toward W, X’s decision is revealed to Y, Y makes a decision toward X, and so on (also see Supplementary Figs. S3 and S4). We allocated the positions in the chain to the participants randomly in advance under the constraint that the participants were never paired with same partners as both upstream and downstream neighbors more than once in a single chain. This procedure was to exclude direct reciprocity. Each participant received a name composed of three randomly selected letters as shown in Fig. 1. The names of the participants near the focal participant in the chain were displayed on the screen of the focal participant (Supplementary Fig. S1). Once a participant submitted a decision, he/she received a new three-letter name, which was different from what he/she had previously received. In addition, we simultaneously ran five chains of decision, and each participant appeared in different chains. Although each participant never paired with same neighbors within each chain, they may play with the same players in a different chain. To assure anonymity, each participant also received different three-letter names in different chains (see Supplementary Method for details). Aside from the three-letter names, each participant was assigned a 7-digit identification that did not change throughout the experiment. The history of decisions of each participant was tracked with his/her 7-digit number, and the payment to each participant was calculated. We ran five chains to accelerate the experiments and prevent the participants from getting tired in later rounds.

For example, in a group of 19 participants, each participant submitted decisions nine times, each toward a different peer, in each of simultaneously running chains. When all participants finished nine decisions in each chain, the game (either the pay-it-forward or reputation-based game) terminated. Thus, each participant made decisions nine times per chain. We defined the round as the number of decisions that a participant made in each type of game, regardless of the chain in which the participant responded (Supplementary Fig. S4). For example, if a participant made decisions six times in each of the five chains and was about to make the seventh decision in one of the chains, the participant was in the 31st round. Because we simultaneously ran five chains (see Supplementary method for details), a participant made 45 decisions (i.e., rounds) in each game. In fact, some participants experienced fewer rounds in the case of smaller groups (with 17 participants) and computer troubles. Supplementary Table S1 shows the lengths of decision chains for all groups. Participants were informed of the structure of the simultaneously running chains of decisions before the experiment.

### Measurement of pro-sociality

We quantified pro-sociality of the participants with a social value orientation (SVO)^40^,^41^,^42^. The SVO is known to be correlated with the tendency to cooperate in social dilemma situations^65^. We measured the SVO with the so-called triple dominance method^42^ one or two months prior to the experiment. When participants registered to the participant pool, they were asked to answer SVO questionnaires in addition to personal information such as the gender.

In the questionnaires, the participants were asked to imagine that they would share money with an unknown person who they had not met before and would not meet again in the future. They chose the most preferred option among the following three alternatives, i.e., pro-social, individualistic, and competitive alternatives. Among the three alternatives, the pro-social option provides the largest joint outcome, i.e., the sum of the money that the participant and the unknown person receive (e.g., 500 yen to the self and 500 yen to the partner). The individualistic option provides the largest outcome to the participant (e.g., 550 yen to the self and 300 yen to the partner). The competitive option provides the largest difference between the two persons (e.g., 500 yen to the self and 100 yen to the partner). Each participant answered such questions nine times (see Supplementary method for the values used in the nine questions). Those who selected at least six consistent options were classified as pro-social, individualist, or competitor. Individualists and competitors were combined into one category to be called pro-self participants because of the small sample size of competitor^40^,^41^. In fact, only three among 131 participants were competitors. Eighty and 35 participants were classified as pro-social and pro-self, respectively. Sixteen participants were not classified because they made inconsistent choices or did not answer the questionnaires.

### Conditional and unconditional probabilities of cooperation

We calculated the unconditional and conditional probabilities of cooperation as follows:


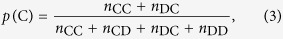



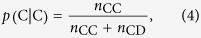



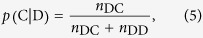


where *n*_CD_ is the total number of defection selected right after the neighbor’s cooperation. *n*_CC_, *n*_DC_, and *n*_DD_ are similarly defined. We calculated *p*(C), *p*(C|C), and *p*(C|D) by aggregating the data over the participants and/or rounds depending on the analysis.

### GLMM

We conducted all analysis using R 3.0.2. Each participant belonged to a group of 17 or 19 persons, and submitted their decisions many times. Decisions by the same participant are not independent. In order to statistically adjust for the effect of these repeated measures, we needed to consider a hierarchical model for analysis. We conducted analysis using a GLMM by using the glmer function implemented in package lme4 in R 3.0.2. In the GLMM analysis, we added a random effect for each participant affecting the intercept. An example of a GLMM logistic regression model with *m* independent variables is given by


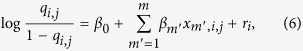


where *i* reperesents the participant [1–131], *j* represents the round [1–45], and *q*_*i,j*_ is a probability that a participant *i* cooperates in the in the *j*th round. *x*_*m’,i,j*_ is the value of an independent variable, β_*m*’_ is the coefficient for the fixed effect of the independent variable, β_0_ is an intercept, and *r*_*i*_ represents the random effect nested within each participant. The random effect was assumed to be normally distributed with mean zero. We estimated β_0_, β_*m*’_, and *r*_*i*_ using the maximum likelihood method assuming the binomial distribution.

### Correlation between conditional probabilities of cooperation in the two games

We calculated the correlation between *p*(C|C) in the two games with the effect of *p*(C) controlled as follows. First, we linearly regressed *p*(C) in the pay-it-forward game on *p*(C|C) in the pay-it-forward game and calculated the residual for each individual. Second, we regressed *p*(C) in the reputation-based game on *p*(C|C) in the reputation-based game and calculated the residual. The residual value represents the tendency of cooperation that is not predicted by unconditional cooperation. Third, we calculated the correlation coefficient between the residual values in the two games by regarding that a participant represents a data point. The correlation coefficient between *p*(C|D) in the two games was similarly calculated.

## Additional Information

**How to cite this article**: Horita, Y. *et al.* Transient nature of cooperation by pay-it-forward reciprocity. *Sci. Rep.* **6**, 19471; doi: 10.1038/srep19471 (2016).

## Supplementary Material

Supplementary Information

## Figures and Tables

**Table 1:** Results of the multivariate GLMM analysis separately for each type of game with a two-way interaction term.

	Odds ratio	95 % CI	P-value
Pay-it-forward			
Decision of the previous participant (D = 0, C = 1)	1.290	0.939 – 1.771	0.116
SVO (pro-self = 0, pro-social = 1)	7.510	2.035 – 27.716	0.002
Gender (female = 0, male = 1)	0.776	0.220 – 2.736	0.693
Round	0.977	0.968 – 0.985	0.000
Decision of the previous participant × Round	0.986	0.974 – 0.998	0.024
Intercept	0.200	0.063 – 0.635	0.006
Reputation-based			
Decision of the previous participant (D = 0, C = 1)	8.481	5.913 – 12.164	<0.001
SVO (pro-self = 0, pro-social = 1)	1.290	0.384 – 4.338	0.681
Gender (female = 0, male = 1)	2.863	0.872 – 9.397	0.083
Round	0.998	0.988 – 1.009	0.777
Decision of the previous participant × Round	0.993	0.979 – 1.007	0.333
Intercept	0.482	0.164 – 1.419	0.186

The dependent variable was the participant’s decision (0 = D, 1 = C). We excluded the participants whose pro-sociality could not be classified and those who did not report the gender. We confirmed that all VIF values were less than 10.
