# Supplementary material for: Transient nature of cooperation by pay-it-forward reciprocity
Source: Sci Rep. 2016 Jan 20;6:19471. doi: 10.1038/srep19471 (PMC4726336; doi:10.1038/srep19471)
Supplement: Supplementary Information [file srep19471-s1.pdf]

Supplementary Information
for "Transient nature of cooperation by pay-it-forward reciprocity"
by Horita, Takezawa, Kinjo, Nakawake, and Masuda

Supplementary Figures
- Figure S1: Exemplar decision screens
- Figure S2: Relationship between R (= p(C|C) − p(C|D)) in the two types of game
- Figure S3: Schematic of chains of decisions consisting of five participants
- Figure S4: Schematic of two simultaneously running chains
- Figure S5: Schematic of the two-state Markov chain model

Supplementary Tables
- Table S1: Statistics of the groups of participants
- Table S2: Selection of the multivariate GLMM containing up to three-way interaction
- Table S3: Analysis of the best GLMM containing up to three-way interaction
- Table S4: Population averages of p(C|C) and p(C|D) in each round and type of game
- Table S5: Univariate GLMM analysis
- Table S6: Multivariate GLMM analysis when both the individual and the group were added as random effects
- Table S7: Multivariate GLMM analysis in the pay-it-forward game with an additional interaction term, i.e., interaction between the decision of the previous participant and the SVO

Supplementary Methods
- Running chains of decisions
- Questionnaire used for measuring social value orientation
- Maximum likelihood estimate for the two-state Markov chain

Supplementary Figures

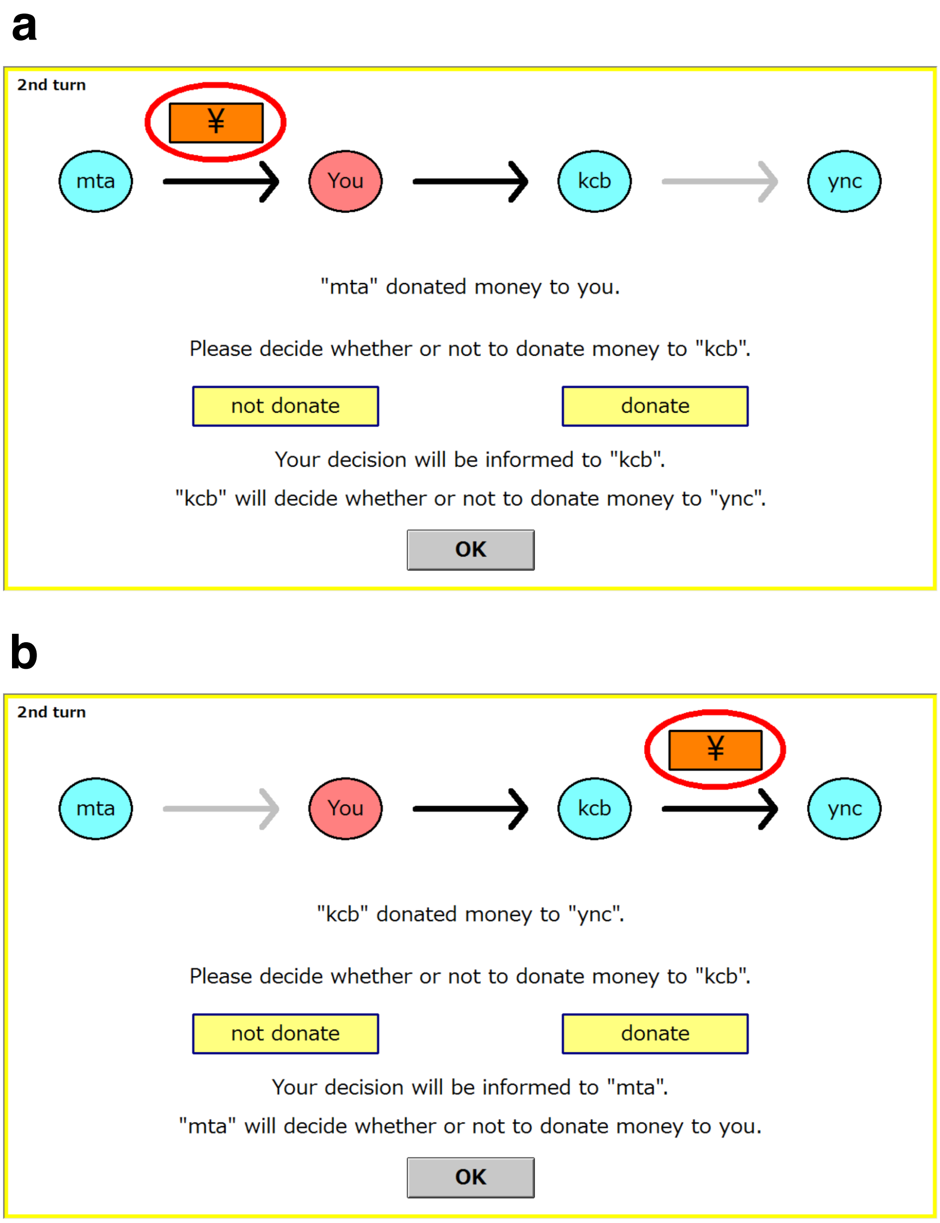

Supplementary Figure S1: Exemplar decision screens. (a) Pay-it-forward game, (b) Reputation-based game. The captions displayed to the participants were written in Japanese. Here we translated them into English.

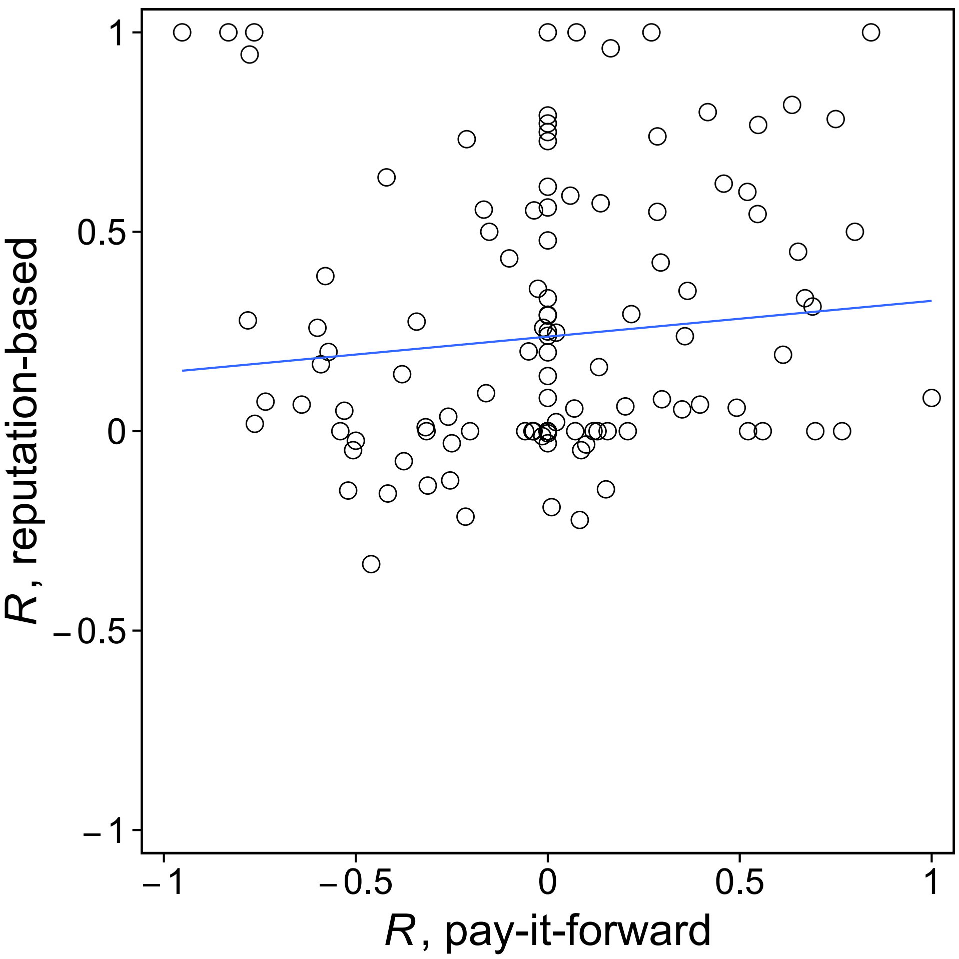

Supplementary Figure S2: Relationship between R (= p(C|C) − p(C|D)) in the two types of game. A circle represents a participant. The line represents the linear regression. Many circles are located on line $x = 0$ or $y = 0$ and overlap with each other because of substantial fractions of unconditional cooperators and unconditional defectors.

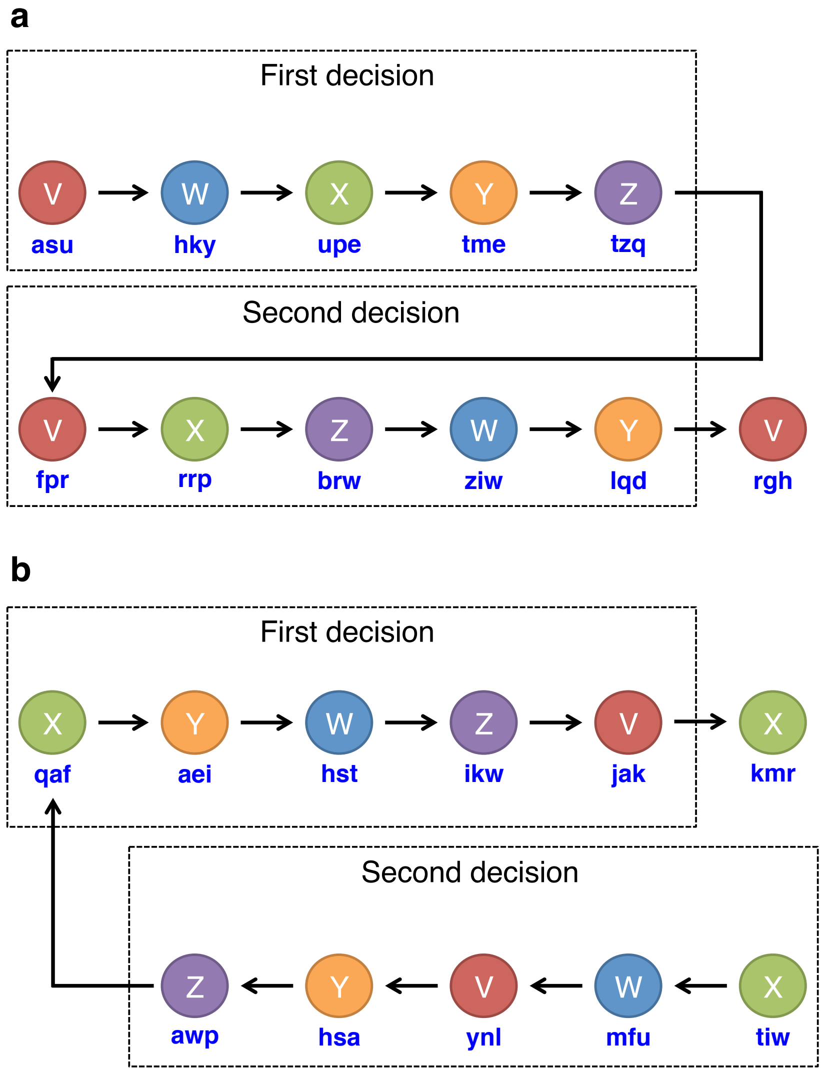

Supplementary Figure S3: Schematic of chains of decisions consisting of five participants. (a) Pay-it-forward game. (b) Reputation-based game.

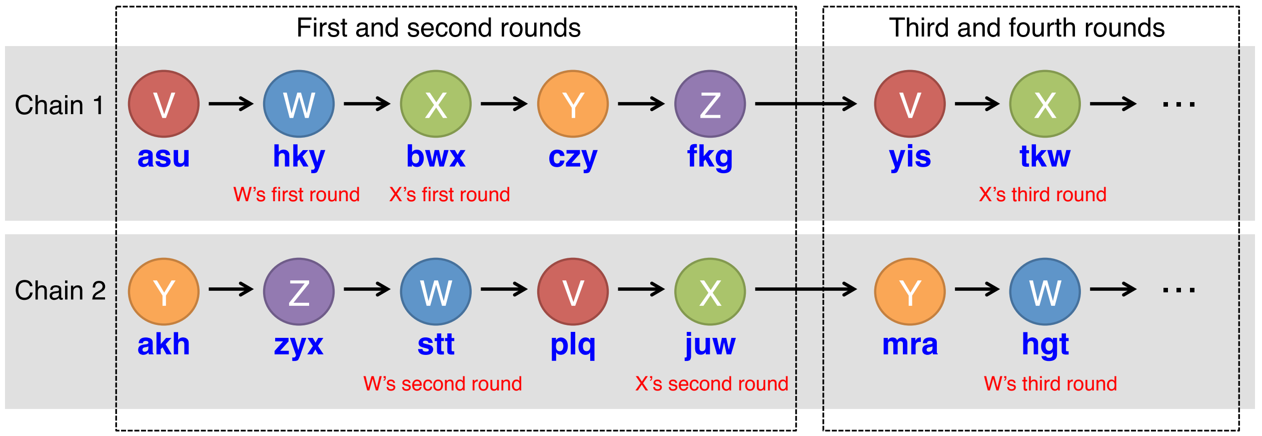

Supplementary Figure S4: Schematic of two simultaneously running chains. The case of the pay-it-forward game is shown for expository purposes.

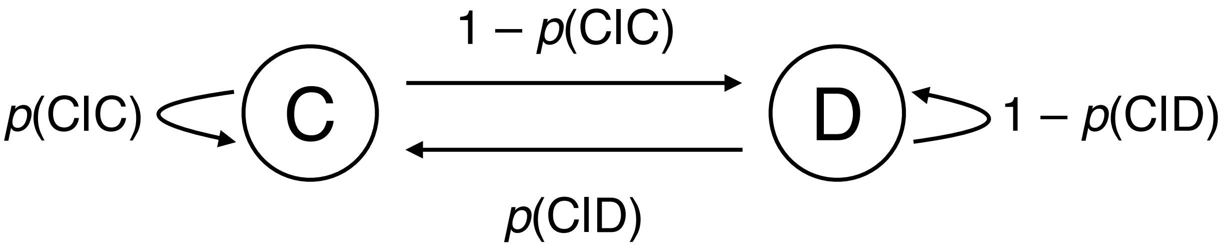

Supplementary Figure S5: Schematic of the two-state Markov chain model.

Supplementary Tables

Supplementary Table S1: Statistics of the groups of participants. In group 4, one participant did not report his/her gender when registering to the participant pool. In the row "order of games", P-R indicates that the participants first played the pay-it-forward game and then the reputation-based game. R-P indicates the opposite order of the games.

Group	1	2	3	4	5	6	7
Number of participants	19	19	17	19	19	19	19
Male	5	7	7	4	7	8	9
Female	14	12	10	14	12	11	10
Cooperator	14	11	7	13	13	9	13
Individualist	4	7	5	2	5	7	2
Competitor	0	0	1	1	1	0	0
Unclassified	1	1	4	3	0	3	4
p(C), pay-it-forward	0.56	0.21	0.57	0.40	0.38	0.33	0.51
p(C), reputation-based	0.65	0.51	0.70	0.76	0.62	0.57	0.51
Chain length, pay-it-forward	171	171	136	171	171	171	171
Chain length, reputation-based	114	171	136	62	171	171	171
Order of games	P-R	R-P	P-R	R-P	P-R	R-P	P-R

Supplementary Table S2: Selection of the multivariate GLMM containing up to three-way interaction.

We carried out GLMM analysis including up to the three-way interaction term, “DPP x round x game type”, where DPP stands for the decision of the previous participant. The independent variables were the decision of the previous participant (D = 0, C = 1), SVO (pro-self = 0, pro-social = 1), round (integer valued, ranging from 1 to 45), gender (female = 0, male = 1), and game type (reputation-based game = 0, pay-it-forward game = 1). The dependent variable was the participant’s decision (D = 0, C = 1). We examined 16 models in which each of the four possible two- or three-way interaction terms were switched on or off. The four possible interaction terms were “DPP x round”, “DPP x game type”, “round x game type”, and “DPP x round x game type”. In the table, the interaction terms added and not added to the GLMM are represented by 1 and 0, respectively. For each estimated GLMM, we calculated the Akaike’s information criterion (AIC). We found that model 9, which did not include “DPP x round” and did include the other two two-way interaction terms and the sole three-way interaction term, realized the smallest AIC value.

Model	DPP x Round	DPP x Game type	Round x Game type	DPP x Round x Game type	AIC
Model 1	1	1	1	1	9037.2
Model 2	1	1	1	0	9036.6
Model 3	1	1	0	1	9041.8
Model 4	1	1	0	0	9058.7
Model 5	1	0	1	1	9068.7
Model 6	1	0	1	0	9216.0
Model 7	1	0	0	1	9069.0
Model 8	1	0	0	0	9231.8
Model 9	0	1	1	1	9035.7
Model 10	0	1	1	0	9039.8
Model 11	0	1	0	1	9041.0
Model 12	0	1	0	0	9059.1
Model 13	0	0	1	1	9081.0
Model 14	0	0	1	0	9220.1
Model 15	0	0	0	1	9079.1
Model 16	0	0	0	0	9233.5

Supplementary Table S3: Analysis of the best GLMM containing up to three-way interaction. We analyzed model 9 in Supplementary Table S2. The dependent variable was the participant's decision (D = 0, C = 1). We confirmed that all VIF values were less than 10.

	Odds ratio	95 % CI	P-value
Decision of the previous participant (D = 0, C = 1)	4.739	4.040 – 5.559	< 0.001
SVO (pro-self = 0, pro-social = 1)	2.414	0.981 – 5.939	0.055
Gender (female = 0, male = 1)	1.377	0.572 – 3.314	0.476
Round	0.994	0.988 – 1.000	0.071
Game type (reputation-based = 0, pay-it-forward = 1)	0.996	0.763 – 1.299	0.976
Decision of the previous participant x Game type	0.314	0.226 – 0.436	<0.001
Round x Game type	0.987	0.977 – 0.996	0.007
Decision of the previous participant x Round x Game type	0.986	0.974 – 0.997	0.013
Intercept	0.444	0.200 – 0.986	0.046

Supplementary Table S4: Population averages of p(C|C) and p(C|D) in each round and type of game. The reported P-values were calculated on the basis of the Fisher's exact test comparing p(C|C) and p(C|D) within each game.

	Pay-it-forward			Reputation-based		
Round	p(C|C)	p(C|D)	P-value	p(C|C)	p(C|D)	P-value
1	0.700	0.444	0.018	0.847	0.458	0.000
2	0.587	0.375	0.022	0.750	0.486	0.006
3	0.557	0.529	0.861	0.774	0.404	0.000
4	0.597	0.492	0.290	0.692	0.491	0.028
5	0.550	0.380	0.056	0.667	0.434	0.012
6	0.419	0.449	0.860	0.795	0.333	0.000
7	0.507	0.391	0.220	0.694	0.478	0.023
8	0.509	0.446	0.486	0.704	0.440	0.003
9	0.500	0.451	0.602	0.743	0.421	0.000
10	0.466	0.452	1.000	0.639	0.424	0.022
11	0.433	0.451	0.862	0.667	0.500	0.068
12	0.500	0.423	0.386	0.786	0.383	0.000
13	0.365	0.456	0.367	0.675	0.521	0.095
14	0.350	0.535	0.037	0.675	0.500	0.048
15	0.431	0.411	0.860	0.738	0.471	0.003
16	0.509	0.419	0.377	0.680	0.547	0.141
17	0.456	0.392	0.480	0.750	0.590	0.090
18	0.429	0.390	0.715	0.700	0.455	0.011
19	0.423	0.494	0.477	0.705	0.373	0.001
20	0.387	0.391	1.000	0.681	0.450	0.026
21	0.322	0.500	0.051	0.706	0.455	0.010
22	0.411	0.373	0.719	0.694	0.480	0.033
23	0.414	0.411	1.000	0.667	0.462	0.036
24	0.404	0.468	0.479	0.657	0.400	0.011
25	0.400	0.432	0.856	0.618	0.523	0.335
26	0.396	0.423	0.857	0.685	0.385	0.003
27	0.347	0.378	0.852	0.619	0.469	0.129
28	0.360	0.432	0.466	0.698	0.469	0.020
29	0.271	0.349	0.438	0.729	0.500	0.024
30	0.295	0.379	0.439	0.619	0.408	0.036
31	0.434	0.359	0.466	0.700	0.442	0.020
32	0.370	0.388	0.853	0.565	0.468	0.409
33	0.318	0.437	0.257	0.750	0.444	0.003
34	0.353	0.400	0.713	0.662	0.321	0.003
35	0.357	0.347	1.000	0.673	0.386	0.007
36	0.444	0.384	0.575	0.685	0.385	0.006
37	0.500	0.342	0.101	0.549	0.476	0.536
38	0.397	0.315	0.361	0.776	0.432	0.001
39	0.370	0.400	0.851	0.685	0.487	0.085
40	0.500	0.321	0.045	0.617	0.485	0.275
41	0.317	0.356	0.837	0.750	0.406	0.004
42	0.400	0.342	0.673	0.714	0.333	0.002
43	0.349	0.352	1.000	0.721	0.303	0.000
44	0.326	0.282	0.675	0.780	0.400	0.001
45	0.324	0.351	0.836	0.514	0.308	0.102

Supplementary Table S5: Univariate GLMM analysis. The dependent variable is the participant's decision (D = 0, C = 1).

	Odds Ratio	95 % CI	P-value
Pay-it-forward			
Decision of the previous participant (D = 0, C = 1)	0.991	0.856 – 1.148	0.908
SVO (pro-self = 0, pro-social = 1)	6.973	1.946 – 24.982	0.003
Gender (female = 0, male = 1)	1.317	0.401 – 4.321	0.650
Round	0.972	0.967 – 0.978	< 0.001
Reputation-based			
Decision of the previous participant (D = 0, C = 1)	7.541	6.287 – 9.046	< 0.001
SVO (pro-self = 0, pro-social = 1)	1.373	0.462 – 4.087	0.568
Gender (female = 0, male = 1)	3.141	1.177 – 8.387	0.022
Round	0.992	0.986 – 0.998	0.008

Table S6: Multivariate GLMM analysis when both the individual and the group were added as random effects. Each type of game was separately analyzed. The dependent variable was the participant's decision (D = 0, C = 1). We confirmed that all VIF values were less than 10.

	Odds ratio	95 % CI	P-value
Pay-it-forward			
Decision of the previous participant (D = 0, C = 1)	1.282	0.933 – 1.761	0.126
SVO (pro-self = 0, pro-social = 1)	6.596	1.821 – 23.895	0.004
Gender (female = 0, male = 1)	0.892	0.261 – 3.049	0.855
Round	0.977	0.968 – 0.985	<0.001
Decision of the previous participant x Round	0.986	0.974 – 0.998	0.025
Intercept	0.214	0.058 – 0.783	0.020
Reputation-based			
Decision of the previous participant (D = 0, C = 1)	8.478	5.910 – 12.160	< 0.001
SVO (pro-self = 0, pro-social = 1)	1.256	0.368 – 4.282	0.716
Gender (female = 0, male = 1)	2.980	0.887 – 10.010	0.077
Round	0.999	0.988 – 1.009	0.790
Decision of the previous participant x Round	0.993	0.979 – 1.007	0.332
Intercept	0.486	0.161 – 1.469	0.201

Table S7: Multivariate GLMM analysis in the pay-it-forward game with an additional interaction term, i.e., interaction between the decision of the previous participant and the SVO. The dependent variable was the participant's decision (D = 0, C = 1). We confirmed that all VIF values were less than 10.

	Odds ratio	95 % CI	P-value
Decision of the previous participant (D = 0, C = 1)	1.007	0.658 – 1.541	0.975
SVO (pro-self = 0, pro-social = 1)	6.591	1.765 – 24.605	0.005
Gender (female = 0, male = 1)	0.775	0.219 – 2.739	0.692
Round	0.976	0.968 – 0.985	<0.001
Decision of the previous participant x Round	0.985	0.973 – 0.998	0.021
Decision of the previous participant x SVO	1.395	0.952 – 2.044	0.088
Intercept	0.221	0.069 – 0.706	0.011

Supplementary Methods

Running chains of decisions

In each type of game, the participants were embedded in chains and made decisions about 40 times. A schematic of a group of five participants engaged in the pay-it-forward reciprocity game is shown in Supplementary Fig. S3(a). Player V, W, X, Y, and Z made decisions in this order. It should be noted that, to make a decision, W referred to the decision that V had made toward W. Similarly for X, Y, and Z. When Z's decision finished, the positions of the five participants were shuffled such that the upstream and downstream neighbors of each participant were those who the participant had not interacted in the first decision. For example, W's neighbors were V and X in the first decision and Y and Z in the second decision. This treatment excludes possibilities of direct reciprocity, which would occur when the same players meet repeatedly. We assumed that the first player was always the same right after shuffling (i.e., V in Fig. S3(a)). However, this assumption was immaterial. Then, the five participants sequentially made their second decisions. Then, the positions of the participants were reshuffled such that each participant avoided anybody that the participant had as a neighbor previously. We continued the decisions and shuffling until we exhausted all possible participants as neighbors. Because each decision consumed two other participants as neighbors of a given participant, there were T decision opportunities for a group of $2T+1$ participants in one chain.

Participants were assigned computer-generated random three-letter names, which were displayed on their screens (see Supplementary Fig. S1). Prior to each decision, the participants received new and unique three-letter names. In Supplementary Fig. S3(a), player W received name "hky" in the first decision and "ziw" in the second decision. This treatment intended to make participants feel that they were interacting with strangers in every decision.

Supplementary Fig. S3(b) schematically depicts a chain of decisions with five participants engaged in the reputation-based game. First, V made a decision toward X. It should be noted that V did not refer to X's previous decision, which did not exist at this stage. Therefore, we excluded V's first decision from the analysis. Second, Z made a decision toward V, referring to the decision that V had made toward X. Similarly for W, Y, and X. When X's decision finished, the positions of the five participants were randomly shuffled in the same way as in the pay-it-forward game. Then, the participants sequentially made the second decision. We continued the decisions and shuffling until we exhausted all possible participants as neighbors. As in the pay-it-forward game, the participants received computer-generated unique three-letter names prior to each decision.

One group consisted of 17 or 19 participants. Therefore, if a single chain were run as we explained with Supplementary Fig. S3, a participant would have to wait for 16 or 18 participants' decisions until the next decision opportunity on average. The mean response time was equal to 3.99 sec, which would lead to the mean waiting time of 3.99 x 16 = 63.84 sec. Then, the participants would easily get bored. Therefore, we simultaneously ran multiple chains of decisions. If the decision opportunity arrived at a participant too frequently, the participant would have doubted that he/she was embedded in the chain as a natural interpretation of the instructions would stand.

Therefore, we simultaneously ran five chains as follows. Here, we explain a simplified case of two chains and a group of five players (Supplementary Fig. S4). Initially, the five participants were randomly ordered in each of the two chains. In this example, "asu" and "akh" first made

decisions in chains 1 and 2, respectively. When both "asu" and "akh" submitted the decisions, the decision screens were simultaneously displayed to the next player in each chain, i.e.,"hky" and "zyx". We repeated this procedure. We assured that the same player did not appear in different chains at the same time. When the fifth participant submitted the decision in both chains, two rounds were completed. It should be noted that the round was defined as the number of decisions that a participant made regardless of the chain in which the participant responded. In Supplementary Fig. S4, two participants simultaneously making decisions in the different chains may be in different rounds. For example, X and W under the anonymous names "bwx" and "stt" in Supplementary Fig. S4 are in their first and second rounds, respectively.

As mentioned above, a player never saw the same neighbor within each chain. However, they may have played with same participants in different chains. This fact was not problematic because each participant received different three-letter names in different chains and rounds. Prior to the experiment, participants were informed of the structure of the simultaneously running chains of decisions.

In a group of 19 participants, a participant made decisions nine times in a chain. When no failure occurred, the length of a chain was equal to 171 (= 19 participants x 9 decisions), and each participant submitted 45 decisions (= 9 decisions x 5 chains) in each type of game.

<u>Questionnaire used for measuring social value orientation</u>

Question 1:
A: You get 480 yen, and the partner gets 80 yen.
B: You get 540 yen, and the partner gets 280 yen.
C: You get 480 yen, and the partner gets 480 yen.

Question 2.
A: You get 560 yen, and the partner gets 300 yen.
B: You get 500 yen, and the partner gets 500 yen.
C: You get 500 yen, and the partner gets 100 yen.

Question 3.
A: You get 520 yen, and the partner gets 520 yen.
B: You get 520 yen, and the partner gets 120 yen.
C: You get 580 yen, and the partner gets 320 yen.

Question 4.
A: You get 500 yen, and the partner gets 100 yen.
B: You get 560 yen, and the partner gets 300 yen.
C: You get 490 yen, and the partner gets 490 yen.

Question 5.
A: You get 560 yen, and the partner gets 300 yen.
B: You get 500 yen, and the partner gets 500 yen.
C: You get 490 yen, and the partner gets 90 yen.

Question 6.
A: You get 500 yen, and the partner gets 500 yen.

B: You get 500 yen, and the partner gets 100 yen.
C: You get 570 yen, and the partner gets 300 yen.

Question 7.
A: You get 510 yen, and the partner gets 510 yen.
B: You get 560 yen, and the partner gets 300 yen.
C: You get 510 yen, and the partner gets 110 yen.

Question 8.
A: You get 550 yen, and the partner gets 300 yen.
B: You get 500 yen, and the partner gets 100 yen.
C: You get 500 yen, and the partner gets 500 yen.

Question 9.
A: You get 480 yen, and the partner gets 100 yen.
B: You get 490 yen, and the partner gets 490 yen.
C: You get 540 yen, and the partner gets 300 yen.

Maximum likelihood estimate for the two-state Markov chain

The structure of the two-state Markov chain model is shown in Supplementary Fig. S5. The likelihood, denoted by L, with which a quadruple (n_{CC}, n_{CD}, n_{DC}, n_{DD}), where n_{CC} is the number of C after receiving/observing C, n_{CD} is the number of D after receiving/observing C, n_{DC} is the number of C after receiving/observing D, and n_{DD} is the number of D after receiving/observing D, is given by

$$L = p(\mathrm{C}\,|\,\mathrm{C})^{n_{\mathrm{CC}}}[1 - p(\mathrm{C}\,|\,\mathrm{C})]^{n_{\mathrm{CD}}}\, p(\mathrm{C}\,|\,\mathrm{D})^{n_{\mathrm{DC}}}[1 - p(\mathrm{C}\,|\,\mathrm{D})]^{n_{\mathrm{DD}}}. \quad \text{(S1)}$$

The maximum likelihood implies that $\frac{\partial \log L}{\partial p(\mathrm{C}\,|\,\mathrm{C})} = 0$, which yields $p(\mathrm{C}\,|\,\mathrm{C}) = \frac{n_{\mathrm{CC}}}{n_{\mathrm{CC}} + n_{\mathrm{CD}}}$.
Similarly, $\frac{\partial \log L}{\partial p(\mathrm{C}\,|\,\mathrm{D})} = 0$ yields $p(\mathrm{C}\,|\,\mathrm{D}) = \frac{n_{\mathrm{DC}}}{n_{\mathrm{DC}} + n_{\mathrm{DD}}}$. We used the estimated p(C|C) and p(D|D) (= 1 – p(C|D)) as p in equation (2) to derive the theoretical distributions of the length of C and that of D, respectively.
